# Advanced radiomics techniques applied on carotid duplex ultrasound data predict adverse outcomes in acute ischemic stroke

**DOI:** 10.3389/fneur.2026.1878428

**Published:** 2026-08-05

**Authors:** Lianlian Zhang, Haiyan Cao, Lizhu Miao, Xinyuan Zhang, Guofu Shi

**Affiliations:** 1 Department of Ultrasound, The Yancheng Clinical College of Xuzhou Medical University, The First People’s Hospital of Yancheng, Yancheng, Jiangsu, China; 2 Department of Stroke Center, The First Affiliated Hospital of Soochow University, Suzhou, Jiangsu, China; 3 Department of Cardiovascular Medicine, The Sixth Affiliated Hospital of Nantong University, Yancheng Third People’s Hospital, Yancheng, Jiangsu, China

**Keywords:** acute ischemic stroke, carotid atherosclerosis, decision curve analysis, deep learning, prognostic assessment, ultrasound radiomics

## Abstract

**Background:**

Acute ischemic stroke (AIS) is a leading cause of mortality and morbidity worldwide. This study explores the potential of using carotid duplex ultrasound (CDU) radiomics to predict outcomes in AIS patients, enhancing prognostic assessments through advanced imaging technologies.

**Methods:**

We enrolled 105 AIS patients at the Stroke Center of the First Affiliated Hospital of Soochow University. CDU images were obtained and processed using artificial intelligence to extract 1,477 radiomic features. Key features were identified and used to develop a predictive model for patient outcomes 3 months post-stroke.

**Results:**

The developed predictive model demonstrated high accuracy and clinical utility, confirmed by decision curve analysis. Inter-observer reliability was excellent, with a Cohen’s kappa coefficient of 0.93, indicating consistent assessments of plaque vulnerability across different observers.

**Conclusion:**

Carotid duplex ultrasound radiomics significantly enhances the ability to predict adverse outcomes in AIS, offering a precision-based approach that supports personalized management strategies. It could improve clinical outcomes by allowing targeted interventions based on specific radiomic profiles.

## Background

Acute ischemic stroke (AIS) accounts for approximately 80% of all stroke cases (1) and is associated with high rates of disability, mortality, and recurrence (2–4). Accurate prognostic prediction, risk stratification, and effective management are essential for improving secondary stroke prevention. While conventional tools such as the Essen Stroke Risk Score, ABCD2 score, and other prognostic scales remain widely used, the rapid growth of healthcare big data has introduced new opportunities (5). The expanding volume and complexity of medical data have increasingly enabled the application of imaging omics in disease prediction, prognosis, diagnosis, and health management (6–8).

Traditional risk score models in domestic and international AIS studies have made some progress in outcome prediction. However, their accuracy remains limited due to reliance on basic indicators such as age and medical history (8). To address this limitation, our study integrates carotid duplex ultrasound (CDU)-based radiomics with clinical parameters to enhance prognostic precision. Although several models based on clinical factors have been proposed, comprehensive studies incorporating CDU imaging features are still limited (9, 10).

AIS is a multifactorial disease in which immune-inflammatory mechanisms play a critical role (11–13). The systemic immune-inflammation index (SII), a novel marker derived from standard blood counts, offers an integrated view of immune and inflammatory status. While its prognostic value has been established in cardiovascular diseases and malignancies (14–17), its role in AIS remains underexplored. Moreover, accumulating evidence suggests that carotid stenosis severity alone is insufficient to predict stroke risk (18). Instead, plaque vulnerability has emerged as a key determinant of ischemic events. Radiomics has enabled the extraction of subtle image features beyond visual assessment, positioning itself as a valuable tool in AIS research (19, 20). Previous studies have also highlighted the association between carotid plaque imaging characteristics and AIS outcomes (21).

Although high-field MRI (3T/7T)-based radiomics allows high-resolution analysis of carotid plaque composition (22), its clinical applicability remains limited due to high costs and restricted equipment availability. In contrast, CDU offers advantages such as low cost, real-time imaging, and operational convenience, making it more suitable for widespread use in primary care. However, conventional CDU assessments are often subjective and lack standardized quantitative metrics, limiting their role in precision medicine. To address these limitations, we propose an integrated approach that combines radiomic features extracted from CDU with the SI, a blood-derived marker reflecting systemic inflammatory status. Given the time-sensitive nature of AIS management (13), CDU’s rapid acquisition and repeatability make it ideal for real-time monitoring of plaque evolution, potentially compensating for the inefficiencies and limitations of MRI in acute settings.

In parallel, machine learning has shown strong potential in acute disease prediction using routine laboratory data, particularly in acute kidney injury (AKI) (23–25). Similar strategies have been applied to AIS prognostication using common biomarkers such as albumin and urinary creatinine, achieving promising accuracy. Building on this, our study aims to evaluate the feasibility and accuracy of CDU-based radiomic features in identifying plaque vulnerability and to construct a multimodal prediction model by integrating radiomics with SII and other clinical parameters. We further assess its adaptability and translational potential across different healthcare settings. Ultimately, we seek to develop a rapid, scalable, and quantifiable risk stratification tool to support individualized decision-making and improve functional outcomes in AIS patients.

## Methods

### Study population

**Figure 1:**
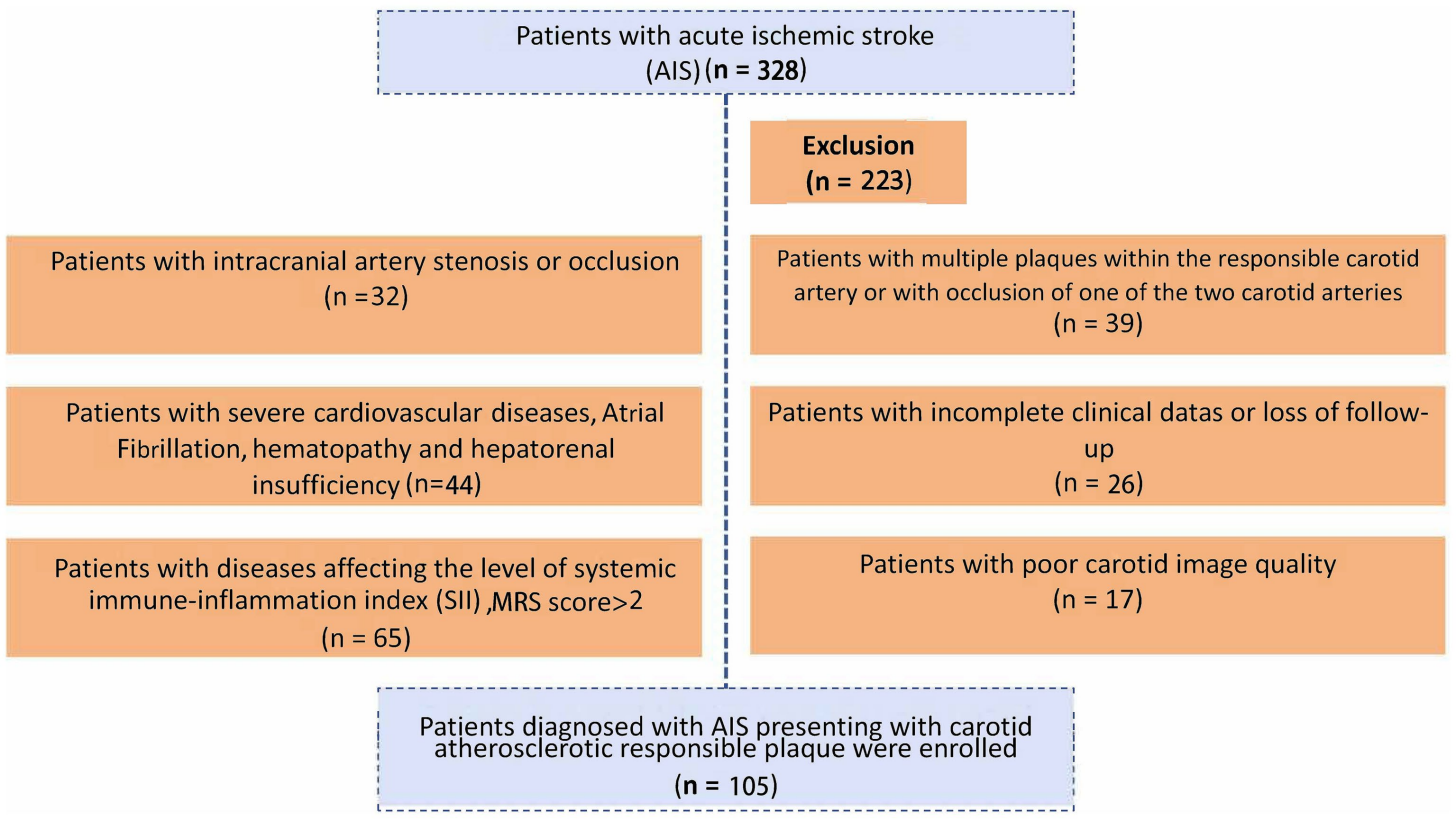
The process of patient inclusion and exclusion.

From January 2018 to December 2020, we prospectively enrolled 105 patients diagnosed with acute ischemic stroke (AIS) associated with carotid atherosclerotic plaques at the Stroke Center of the First Affiliated Hospital of Soochow University. CDU images were obtained for all participants (Figure 1).

Inclusion criteria were as follows: (1) AIS confirmed with atherosclerotic abnormalities in the extracranial segments of the internal or common carotid arteries; (2) age ≥18 years; (3) admission within 3 days of symptom onset; (4) receipt of standard medical treatment; (5) CDU performed within 24 h of admission; (6) availability of three-month follow-up data; and (7) brain MRI with diffusion-weighted imaging (DWI) or CT perfusion performed upon admission with image quality sufficient for infarct volume quantification. Exclusion criteria included: (1) diagnosis of hemorrhagic cerebrovascular disease or intracranial arterial stenosis; (2) atrial fibrillation or other arrhythmias suggestive of cardioembolic stroke; (3) severe cardiovascular, hematologic, hepatic, or renal diseases; (4) concomitant acute infection, sepsis, malignancy, or autoimmune disease; (5) ongoing treatment with immunosuppressants, corticosteroids, or cytotoxic drugs; (6) history of thrombolysis, thrombectomy, carotid endarterectomy, or stenting; (7) absence of carotid plaques or complete occlusion of either the internal or common carotid artery; (8) baseline modified Rankin Scale (mRS) score >2; and (9) incomplete clinical, laboratory, or imaging data, including suboptimal image clarity or missing follow-up.

Patients were categorized into anterior and posterior circulation groups based on TOAST classification and neuroimaging to reduce heterogeneity. The anterior circulation group was defined by infarctions in the MCA or ACA territories on DWI or an ASPECTS score ≤7 on CT; the posterior circulation group was defined by infarctions in the PCA or vertebrobasilar territories on DWI or a PC-ASPECTS score ≤8 on CT. Infarct volumes were manually segmented using ITK-SNAP software on DWI images. CT images were assessed using the Alberta Stroke Program Early CT Score (ASPECTS). Baseline clinical parameters were recorded within 24 h of admission. The systemic immune-inflammation index (SII) was calculated using the formula: SII = Platelet count × Neutrophil count/Lymphocyte count. The primary outcome was the functional status 3 months post-stroke, assessed by trained professionals via standardized telephone interviews using the modified Rankin Scale (mRS). Patients were classified into favorable (mRS 0–2) or unfavorable (mRS ≥ 3) outcome groups. Patients with baseline mRS > 2 were excluded to ensure uniformity in baseline functional status.

**Figure 2:**
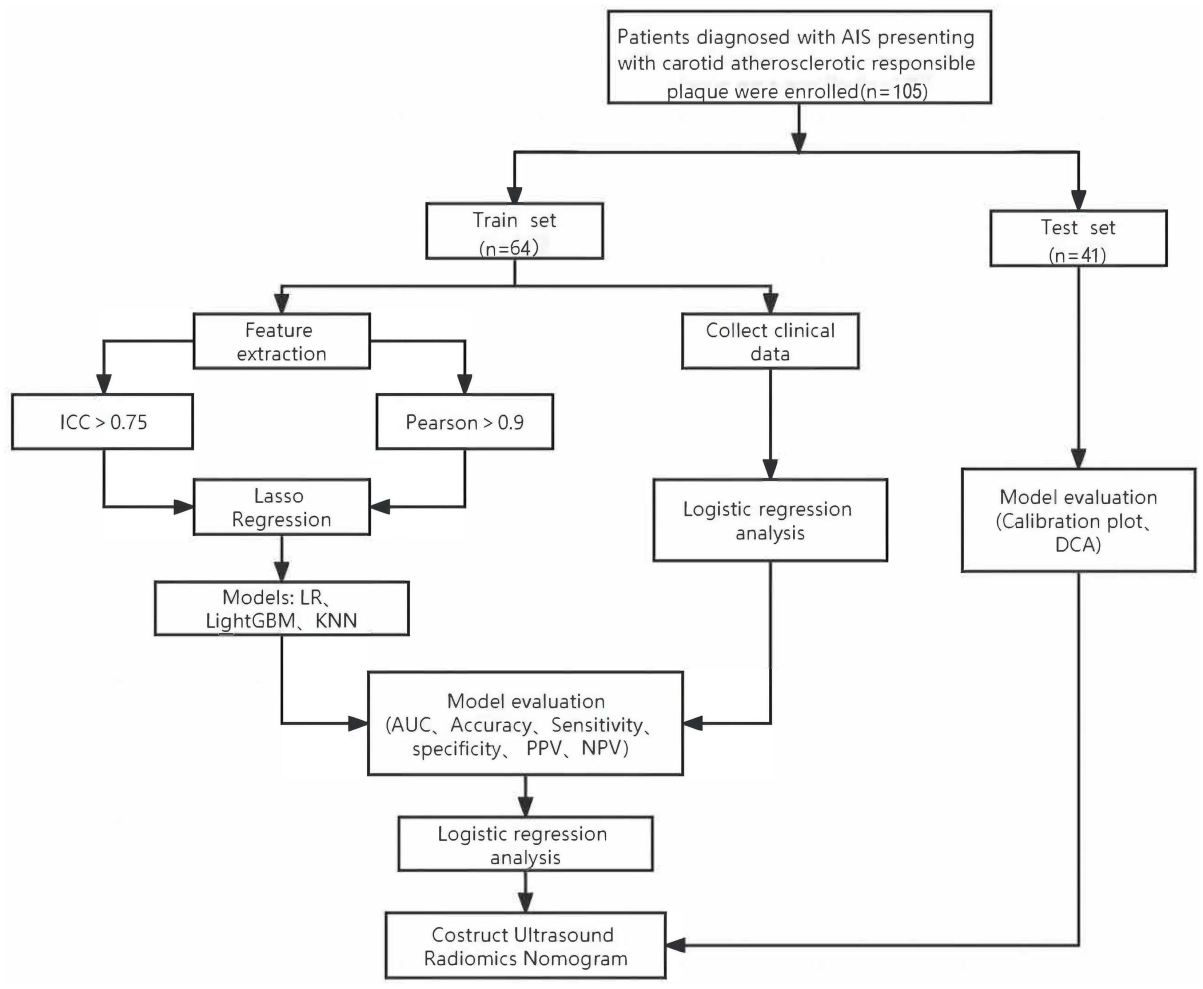
Research workflow diagram.

Additional variables, including age, sex, baseline NIHSS score, and treatment modalities, were collected and adjusted for in multivariate analyses. To control for potential confounding, stratified analyses were conducted based on age (≤65 vs. >65 years), NIHSS score (≤5 vs. >5), and SII (low vs. high) to evaluate the predictive performance of the radiomics model in different subgroups. This study was approved by the Ethics Committee of the First Affiliated Hospital of Soochow University (Approval No. 2011197), and written informed consent was obtained from all participants. The detailed research methodology is depicted in Figure 2.

### CDU protocol

A standardized ultrasound protocol with preset parameters was employed to ensure data accuracy and consistency. CDU examinations were performed using the iU Elite scanner (Philips Healthcare, Netherlands), with L9-3 linear array and C5-1 convex array probes scanning from proximal to distal vascular segments. Transverse and longitudinal views were acquired using grayscale imaging, color flow imaging, and spectral Doppler analysis to assess the bilateral common carotid arteries, carotid artery bifurcation (CAB), and internal carotid arteries (ICA). When plaques were present, their dimensions, morphology, echogenicity, structural characteristics, and degree of vascular stenosis were evaluated from multiple angles and planes. Key hemodynamic parameters were recorded, such as peak systolic velocity, end-diastolic velocity, and resistive index. Images of carotid atherosclerosis (CAS) related plaques were archived for subsequent analysis.

### Analysis of CDU

**Figure 3:**
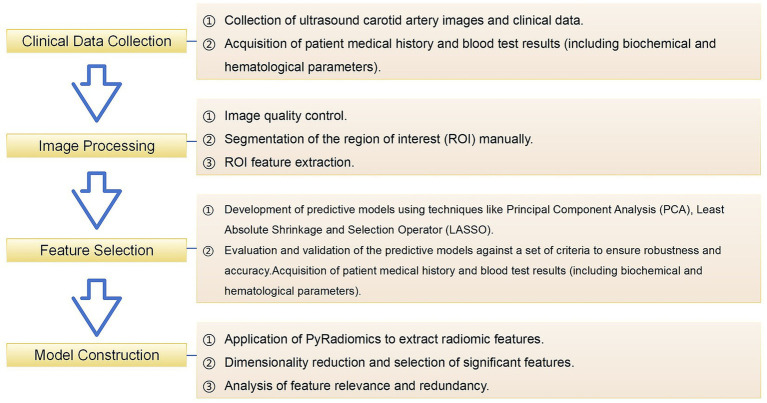
Radiomics feature extraction flowchart.

In this study, the criteria for plaque characterization were based on our team’s previous pathological research. Radiomic features were extracted from CDU images and integrated with clinical data to develop a predictive model. The workflow included feature quality control using the intraclass correlation coefficient (ICC), feature selection via least absolute shrinkage and selection operator (LASSO) regression, and model construction using machine learning algorithms (Figure 3).

Plaques were classified according to a vulnerability score, with a score of 4 indicating vulnerable plaques and scores other than 4 classified as stable. The scoring system was based on ultrasound features such as hypoechogenicity and intraplaque hemorrhage, combined with histological markers like lipid-rich necrotic core and hemorrhage as defined in the literature (26). Two-dimensional ultrasound was used to assess plaque morphology, echogenicity, fibrous cap integrity, and the presence of ulceration (e.g., surface depression >1 mm or visible intraplaque color flow), as well as the degree of stenosis, which was graded from 0 to 3 (27). Due to the limited resolution of CDU in distinguishing lipid core from intraplaque hemorrhage, these features were analyzed as a combined variable (22).

**Figure 4:**
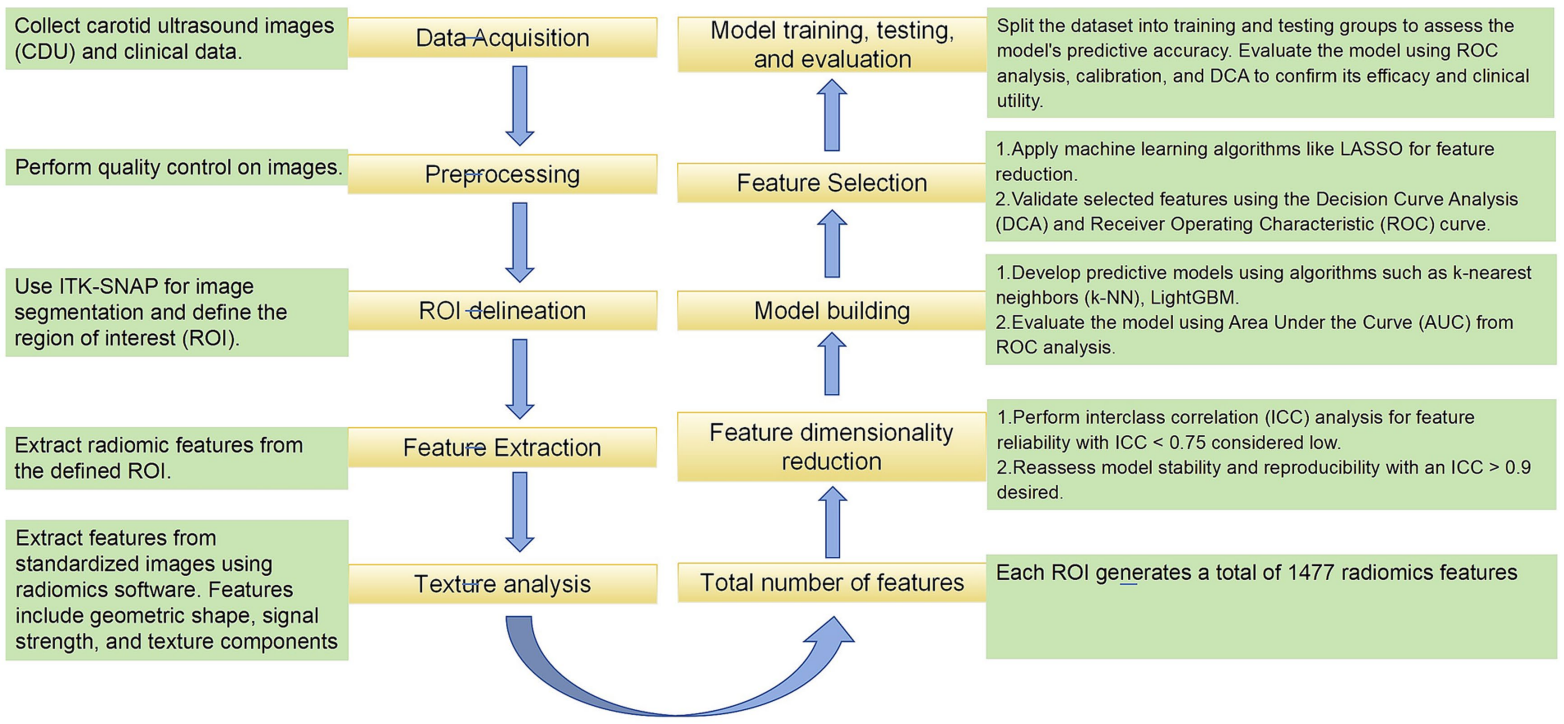
Processing and model building flowchart. This flowchart illustrates the stepwise procedure for selecting key radiomic features from the CDU data and integrating them with clinical factors to construct the predictive model. It encompasses the use of ICC for feature quality control, LASSO regression for feature selection, and the application of machine learning algorithms to establish a prognostic nomogram.

Subsequently, the most prognostically relevant radiomic features were selected to enhance the robustness and clinical applicability of the model. The detailed process is illustrated in the diagram (Figure 4), which outlines the key steps from feature selection to model development, emphasizing the role of statistical and machine learning techniques in processing high-dimensional imaging data to construct a highly accurate and generalizable radiomic nomogram.

### Image segmentation and radiomic feature calculation

**Figure 5:**
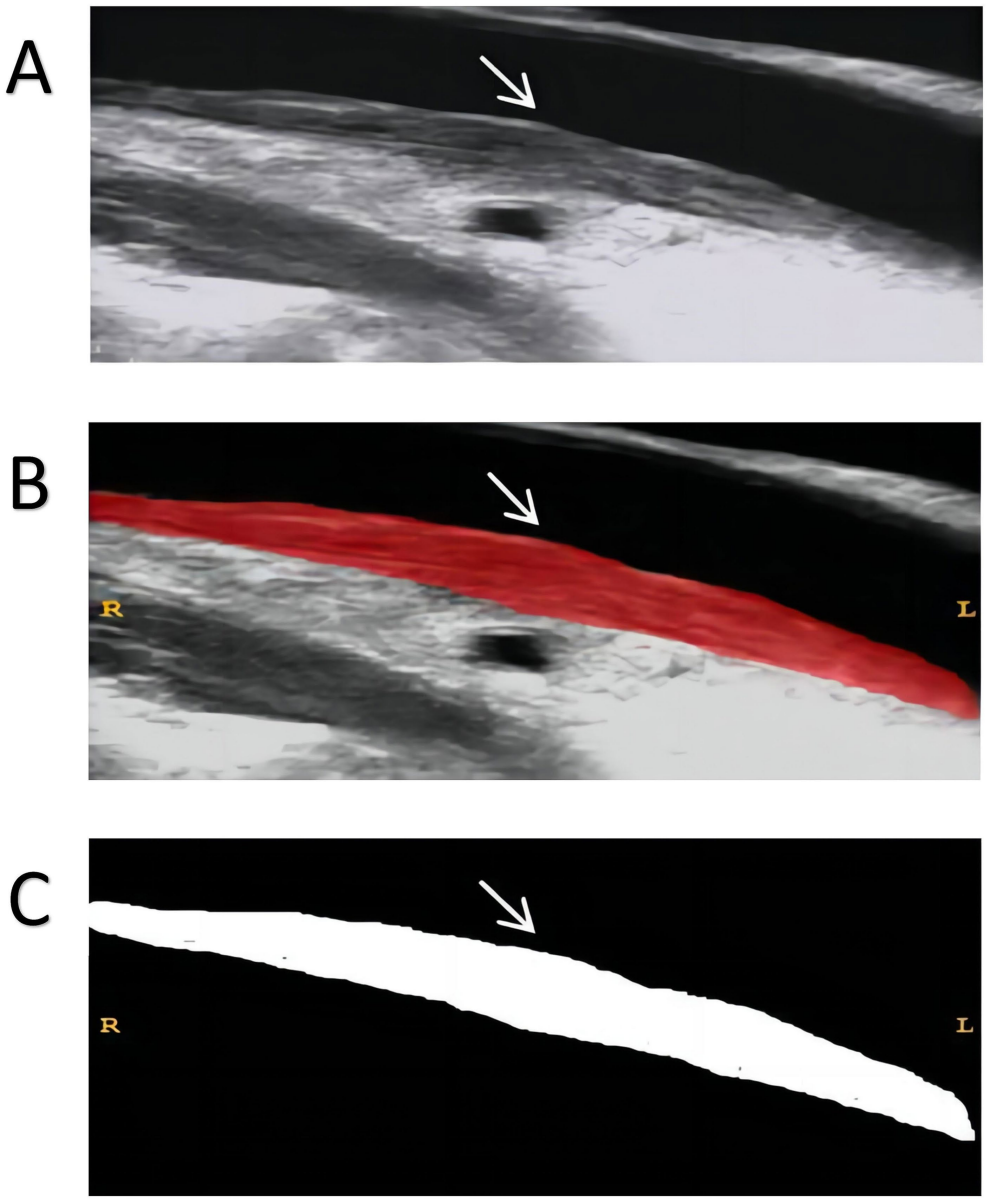
Example of manually segmented CDU images. **(A)** Original ultrasound image of the plaque. **(B)** The segmentation region was adjusted along the maximum longitudinal cross-section of the plaque. **(C)** ITK-SNAP software was used to label the segmented plaque image for further analysis.

The maximum longitudinal section of each carotid plaque was identified during an ultrasound examination based on size, shape, and spatial relationship to surrounding structures, using the iU Elite scanner (Philips Healthcare, Netherlands) for comprehensive multi-angle CDU (carotid duplex ultrasound) scanning. Regions of interest (ROIs) were manually delineated on the maximum longitudinal section using ITK-SNAP 4.0.0-BETA by two experienced radiologists independently and in a blinded manner (Figure 5). Inter-observer agreement was excellent (Cohen’s kappa = 0.93). To minimize variability and ensure feature robustness, a three-step normalization strategy was applied: spatial normalization (resampling to 0.2 × 0.2 mm^2^ resolution), intensity normalization using histogram matching, and morphological compensation by incorporating plaque metrics (length, area, perimeter ratio) as covariates in LASSO regression.

Radiomic features were extracted from both the original CDU images and a series of filtered images. The following image filters were applied: wavelet transform (eight decompositions), Laplacian of Gaussian (LoG) with sigma values of 1.0, 2.0, 3.0, 4.0, and 5.0, Local Binary Patterns (LBP) in 3D, square filter, square root filter, logarithm filter, exponential filter, and gradient filter. From the standardized ROIs (original and filtered images), a total of 1,477 radiomic features were extracted, covering shape, intensity, and texture characteristics. Texture features were derived using methods including gray level co-occurrence matrix (GLCM), gray level run length matrix (GLRLM), gray level size zone matrix (GLSZM), and gray level dependence matrix (GLDM) (28). Highly collinear features (Pearson correlation coefficient > 0.9) were excluded to reduce redundancy. Feature definitions and extraction protocols are available at https://pyradiomics.readthedocs.io/.

### Feature reduction and model development

The entire dataset was first randomly split into a training set (70%, *n* = 74) and a testing set (30%, *n* = 31). All feature reduction and model development procedures were performed exclusively on the training set; the testing set was kept completely independent and was not used until the final model evaluation.

In the training set, radiomic features were preliminarily screened. Features with low reproducibility (intraclass correlation coefficient, ICC < 0.75) and high collinearity (Pearson correlation coefficient > 0.90) were excluded to retain informative and independent variables. Spearman analysis showed only 2 of 1,477 geometric features (equivalent diameter, maximum 3D diameter) were strongly correlated with plaque size (*ρ* > 0.6) and were removed during preprocessing. The remaining features were selected using LASSO regression, with 10-fold cross-validation to determine the optimal penalty. During LASSO cross-validation, feature selection was repeated within each training fold to prevent information leakage from the validation folds. The final model incorporated CDU radiomic features, systemic immune-inflammation index (SII), and clinical variables such as NIHSS score.

Model performance was evaluated using logistic regression (LR), k-nearest neighbor (KNN), and Light Gradient Boosting Machine (LightGBM). LR provides strong interpretability and is suitable for moderate-sized datasets but assumes linear relationships. KNN handles nonlinear data well but is less efficient with large datasets and is sensitive to noise. LightGBM efficiently manages complex, high-dimensional data with automatic feature selection but requires careful tuning to prevent overfitting. The model choice was based on data characteristics and clinical relevance. Performance was assessed via k-fold cross-validation. The dataset was split into k subsets, with each used once as a test set while the others served for training. Metrics such as accuracy and recall were averaged to evaluate model consistency. Discriminative ability was quantified by the area under the ROC curve (AUC), which is robust to class imbalance and widely used in clinical prediction models.

### Validation of the radiomics column chart model

After the feature selection and model building were finalized in the training set, the testing set was used solely for model evaluation. The model was rigorously validated in training and testing sets using ROC, calibration curves, decision curve analysis (DCA), and Brier scores to evaluate discrimination, calibration, and clinical utility. Calibration curves assessed the agreement between predicted and observed outcomes, while Brier scores quantified predictive accuracy by measuring the mean squared difference between predicted probabilities and actual outcomes. Lower Brier scores indicated better calibration, particularly in the testing set, supporting the model’s reliability in clinical applications.

### Statistical analysis

Statistical analysis was performed using SPSS version 26.0 and Python 3.6.7. The dataset was randomly divided into a training set (70%) and a testing set (30%) before any feature selection or model development. All feature reduction steps (ICC, Pearson, LASSO) were performed exclusively on the training set. Normally distributed data were expressed as mean ± standard deviation, and non-normally distributed data as median with interquartile range. Categorical variables were presented as percentages. Group comparisons were conducted using chi-square or Fisher’s exact tests for categorical data, and *t*-tests or Mann–Whitney *U* tests for continuous variables, as appropriate. Univariate and multivariate logistic regression analyses were used to identify independent clinical predictors and to develop the predictive model. Model performance was assessed using ROC curves, calibration curves, and DCA. Inter-rater agreement for image segmentation was evaluated using Cohen’s kappa coefficient. All statistical tests were two-sided, with significance set at *p =* 0.05.

## Results

### Patient characteristics

Participant eligibility was determined based on predefined inclusion and exclusion criteria. One hundred five AIS patients were included, with a mean age of 62.45 ± 10.38 years; 72 were male (68.57%). Among them, 60 patients (57.14%) were diagnosed with hypertension and 47 (44.76%) with diabetes. Baseline characteristics were categorized according to clinical outcomes (favorable vs. unfavorable), as shown in Table 1. Significant differences were observed between the two groups in age, diabetes, NIHSS score, HCY levels, and presence of vulnerable plaques (*p =* 0.05). After excluding patients with atrial fibrillation, radiomic features of carotid plaques remained significantly associated with stroke outcomes (*p =* 0.05). In the anterior circulation subgroup, radiomic features were significantly associated with unfavorable outcomes (*p =* 0.05), while no significant association was observed in the posterior circulation subgroup (*p* = 0.12).

**Table 1:** Baseline data of patients according to poor/good prognosis (*N* = 105).

Characteristics	Poor prognosis (*n* = 78)	Good prognosis (*n* = 27)	*p*-value
Age (median ± SD)	64.23 ± 10.76	62.89 ± 10.14	0.034*
Male (%)	52/26	20/7	0.456
Hypertension (%)	48/30	12/15	0.563
Systolic blood pressure (median ± SD)	136.48 ± 17.12	129.14 ± 14.28	0.065
Diastolic blood pressure (median ± SD)	83.44 ± 10.91	81.73 ± 10.12	0.234
Diabetes mellitus (%)	36/42	11/16	0.045*
Coronary heart disease (%)	38/40	13/14	0.865
Current or former smokers (%)	25/53	12/15	0.340
History of alcohol intake (%)	15/63	5/22	0.567
History statin treatment (%)	24/54	8/19	0.432
History antiplatelet treatment (%)	30/48	9/18	0.125
History of stroke (%)	27/51	7/20	0.456
NIHSS score [Median (Q1 – Q3)]	8.00 [6.00,13.00]	6.40 [4.00,8.50]	0.001*
Fasting blood-glucose (median ± SD)	5.91 [5.00,7.28]	5.88 [5.45,7.15]	0.678
Dyslipidemia (%)	28/50	8/19	0.021*
TC (median ± SD)	4.07 ± 1.04	4.39 ± 1.23	0.567
TG (median ± SD)	1.26 [0.90,1.66]	1.34 [0.92,1.72]	0.530
HDL-C (median ± SD)	1.07 [0.91,1.29]	1.15 [0.97,1.31]	0.165
LDL-C [Median (Q1 – Q3)]	2.52 [1.85,3.15]	2.72 [1.95,3.17]	0.208
Uric acid (median ± SD)	320.25 ± 84.55	310.18 ± 94.33	0.345
Fibrinogen [Median (Q1 – Q3)]	3.21 [2.62,3.79]	3.15 [2.63,4.01]	0.876
HCY (median ± SD)	9.42 [7.48,12.84]	7.95 [5.42,10.44]	0.034*
hs-CRP [Median (Q1 – Q3)]	2.69 [1.72,5.32]	2.70 [1.50,4.85]	0.675
Platelet counts [Median (Q1 – Q3)]	210.50 [172.00,244.75]	193.00 [148.50,235.00]	0.076
Neutrophil counts [Median (Q1 – Q3)]	4.73 [3.65,6.03]	4.39 [3.45,5.36]	0.078
Lymphocyte counts [Median (Q1 – Q3)]	1.68 [1.35,2.15]	1.75 [1.34,2.30]	0.453
Monocyte counts [Median (Q1 – Q3)]	0.53 [0.37,0.62]	0.50 [0.33,0.57]	0.298
SII [Median (Q1 – Q3)]	589.70 [400.82,832.27]	478.50 [295.21,679.79]	0.013*
PLR [Median (Q1 – Q3)]	123.47[96.60,158.82]	114.84 [79.76,138.80]	0.056
MLR [Median (Q1 – Q3)]	0.28 [0.19,0.40]	0.26 [0.18,0.37]	0.132
NLR [Median (Q1 – Q3)]	2.70 [1.96,3.96]	2.35 [1.68,3.44]	0.102
Vulnerable Plaque (%)	52/26	12/15	<0.001*
Ulcerative plaque	50/28	9/18	<0.001*
Hypoechoic plaque	37/41	8/19	0.897
Irregular plaque	49/29	10/17	<0.001*
Infarct volume (cm^3^)	18.3 ± 12.1	32.7 ± 20.5	<0.001*
ASPECTS scoring	8.2 ± 1.5	5.9 ± 2.3	0.003

* indicates *p*-values indicating statistical significance. TC, total cholesterol; TG, triglycerides; HDL-C, high-density lipoprotein cholesterol; LDL-C, low-density lipoprotein cholesterol; NIHSS, National Institutes of Health Stroke Scale; HCY, homocysteine; hs-CRP, high-sensitive C-reactive protein; SII, systemic immune-inflammation index; PLR, platelet to lymphocyte ratio; MLR, monocyte to lymphocyte ratio; NLR, neutrophil to lymphocyte ratio.

### Comparison of clinical and imaging characteristics between poor prognosis and good prognosis groups

**Figure 6:**
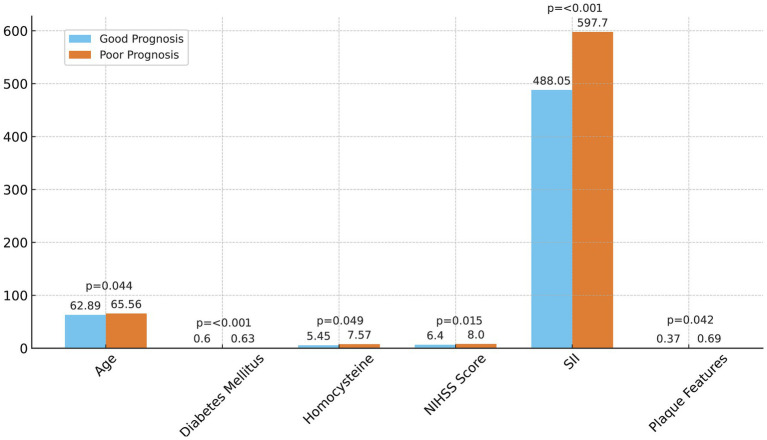
Comparison of clinical and radiological features between poor prognosis group and good prognosis group. The blue bars in the figure represent the good prognosis group, while the orange bars represent the poor prognosis group.

**Figure 7:**
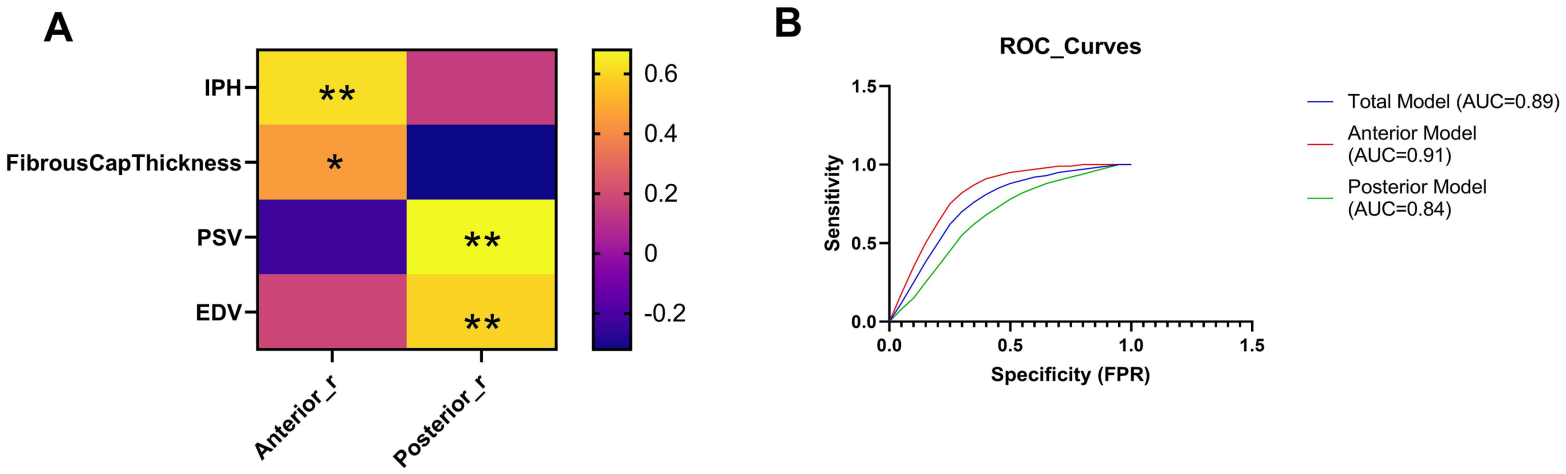
Correlation heatmaps of radiomic features and ROC curve comparisons between anterior and posterior circulation stroke models. **(A)** The heatmap illustrates significant intergroup differences in intraplaque hemorrhage (IPH), fibrous cap thickness, peak systolic velocity (PSV), and end-diastolic velocity (EDV) (*p = 0.05, **p = 0.01). **(B)** ROC curves compare the predictive performance of the overall model, anterior circulation model, and posterior circulation model.

Early identification of prognostic factors is critical in the management of AIS. This study analyzed clinical and biomarker parameters associated with favorable and unfavorable outcomes. Table 1 summarizes the baseline characteristics of 105 AIS patients grouped by outcome. Significant differences were observed in age, diabetes, dyslipidemia, NIHSS score, HCY, SII, and plaque features, including vulnerable, ulcerative, and irregular plaques (*p =* 0.05). As shown in Figure 6, the mean age was higher in the unfavorable group (64.23 years, *n* = 78) than in the favorable group (62.48 years, *n* = 27) (*p* = 0.044). The unfavorable group had a higher prevalence of diabetes (*p* = 0.049) and elevated HCY levels (*p* = 0.042). The median NIHSS score was also higher (8 vs. 6.4, *p* = 0.001), along with SII (*p* = 0.015). Vulnerable, ulcerative, and irregular plaques were significantly more common in the unfavorable group (*p =* 0.001). Subgroup analysis indicated a significant correlation between the radiomic feature GLCM_Contrast and infarct volume in the anterior circulation group (*r* = 0.62, *p =* 0.001), but not in the posterior group (*r* = 0.11, *p* = 0.42). The anterior circulation-specific nomogram achieved an AUC of 0.902 (95% CI: 0.87–0.95), significantly outperforming the unstratified model (ΔAUC = 0.036, *p* = 0.021) (Figure 7).

### Feature selection and radiomics signature development

**Figure 8:**
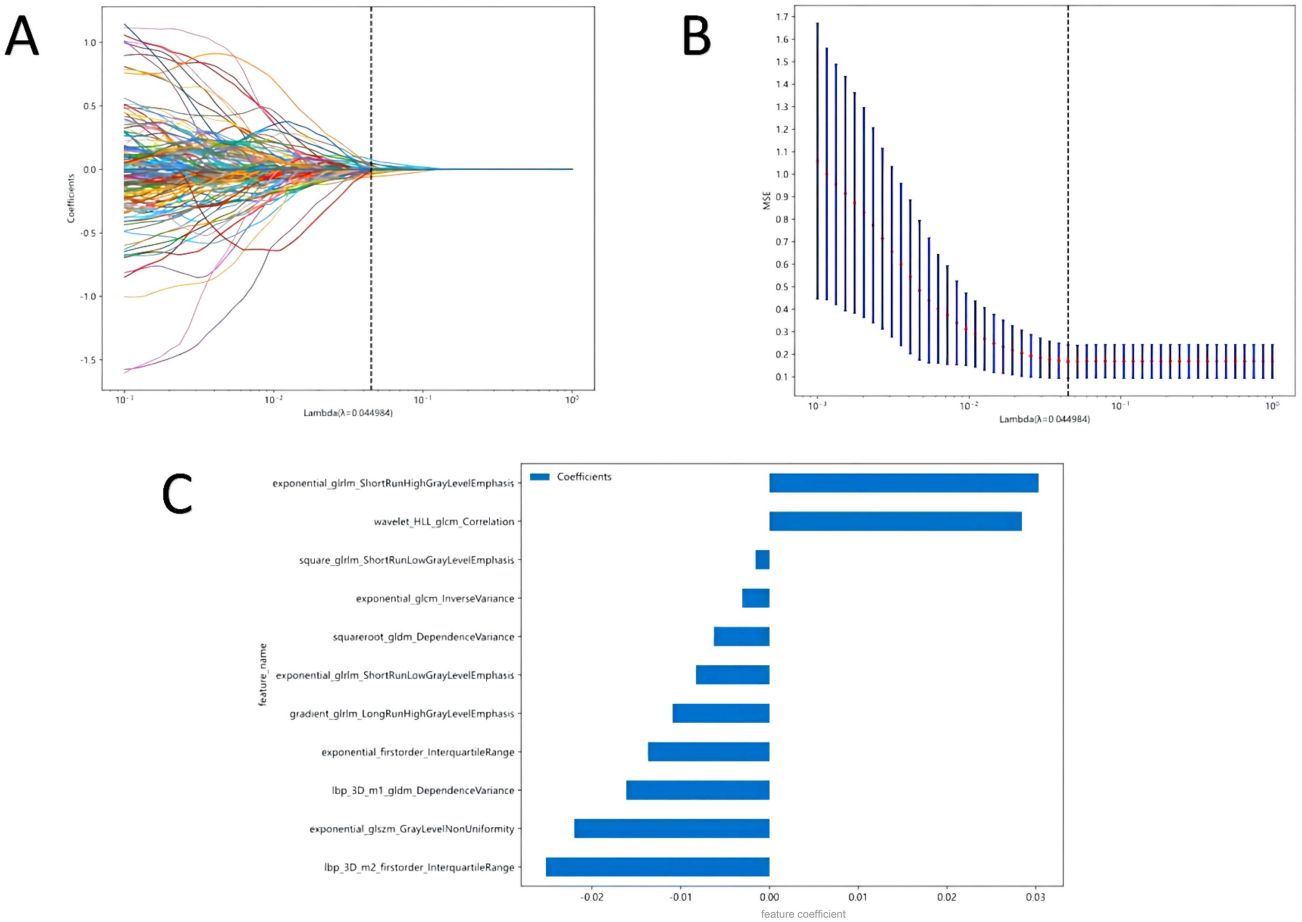
Selection process of radiomics features. **(A)** Variation of variable coefficients with penalty coefficients. **(B)** The optimal penalty coefficient is selected through 10-fold cross-validation. When the binomial deviation is minimized (the minimum standard), 11 optimal radiomics features are chosen. **(C)** Absolute values of the included variable coefficients.

From each plaque, 1,477 ultrasound radiomics features were initially extracted. Following inter-observer consistency evaluation, 88 features with an ICC < 0.75 were excluded. Pearson correlation analysis was then performed to assess feature collinearity, and 1,016 features with Pearson correlation coefficients < 0.9 were retained (Figure 8). Utilizing the intercept and coefficients derived from LR, we established the radiomics scoring formula as follows: Radscore = 0.7883–0.0136exponential_firstorder_InterquartileRange-0.0030exponential_glcm_InverseVariance+0.0303exponential_glrlm_ShortRunHighGrayLevelEmphasis-0.008exponential_glrlm_ShortRunLowGrayLevelEmphasis-0.0219exponential_glszm_GrayLevelNonUniformity-0.0109gradient_glrlm_LongRunHighGrayLevelEmphasis-0.0161lbp_3D_m1_gldm_DependenceVariance-0.0251lbp_3D_m2_firstorder_InterquartileRange-0.0015square_glrlm_ShortRunLowGrayLevelEmphasis-0.0062squareroot_gldm_DependenceVariance+0.0284*wavelet_HLL_glcm_Correlation.

### Multifactorial predictive modeling analysis of adverse outcomes in AIS patients

Univariate and multivariate logistic regression analyses investigated various clinical indicators’ independent contributions and interrelationships in prognostic assessment. As presented in Table 2, univariate analysis revealed that age (OR = 1.284, 95% CI: 1.005–1.399, *p =* 0.045), diabetes mellitus (OR = 1.722, 95% CI: 1.003–2.091, *p =* 0.049), and dyslipidemia (OR = 1.812, 95% CI: 1.019–2.027, *p =* 0.031) were significantly associated with poor prognosis. In multivariate analysis, age remained statistically significant with a slightly lower OR (OR = 1.280, 95% CI: 1.002–1.391, *p =* 0.046).

**Table 2:** Univariate and multivariate logistic regression analysis of factors related to poor prognosis (*N* = 105).

Characteristics	Univariate		Multivariate	
	OR [95% CI]	*p*	OR [95% CI]	*p*
Age	1.284 [1.005–1.399]	0.045*	1.280 [1.002–1.391]	0.046*
Systolic blood pressure	1.015 [0.993–1.036]	0.078	–	–
Diabetes mellitus	1.722 [1.003–2.091]	0.049*	–	–
Dyslipidemia	1.812 [1.019–2.027]	0.031*	–	–
NHISS score	6.102 [2.005–18.800]	0.003*	2.532 [1.901–3.102]	<0.001*
Platelet counts	1.002 [0.999–1.006]	0.088	–	–
Neutrophil counts	1.230 [0.980–1.523]	0.060	–	–
SII	2.952 [2.105–3.620]	0.020*	2.955 [2.251–3.509]	0.017*
PLR	1.006 [1.000–1.013]	0.051*	–	–
Vulnerable Plaque	1.220 [1.110–1.387]	<0.001*	1.225 [1.098–1.379]	<0.001*
Ulcerative plaque	2.174 [2.070–2.420]	<0.001*	–	–
Irregular plaque	1.258 [1.121–1.541]	<0.001*	–	–

* indicates *P*-values indicating statistical significance. OR, Odds ratio; CI, Confidence interval; NIHSS, National Institutes of Health Stroke scale; HCY, Homocysteine; hs-CRP, high-sensitive C-reactive protein; SII, systemic immune-inflammation index.

Furthermore, the NIHSS score and the SII emerged as independent predictors of adverse outcomes in univariate and multivariate analyses. The univariate OR for the NIHSS score was 6.102 (95% CI: 2.005–18.800, *p =* 0.003), and the multivariate OR was 2.532 (95% CI: 1.901–3.102, *p <* 0.001). For the SII, the univariate OR was 2.952 (95% CI: 2.105–3.620, *p =* 0.020), and the multivariate OR was 2.955 (95% CI: 2.251–3.509, *p =* 0.017). Vulnerable plaque also demonstrated significant association with poor prognosis in both analyses, with a univariate OR of 1.220 (95% CI: 1.110–1.387, *p <* 0.001) and a multivariate OR of 1.225 (95% CI: 1.098–1.379, *p <* 0.001).

### Construction of ultrasound radiomics column chart

**Figure 9:**
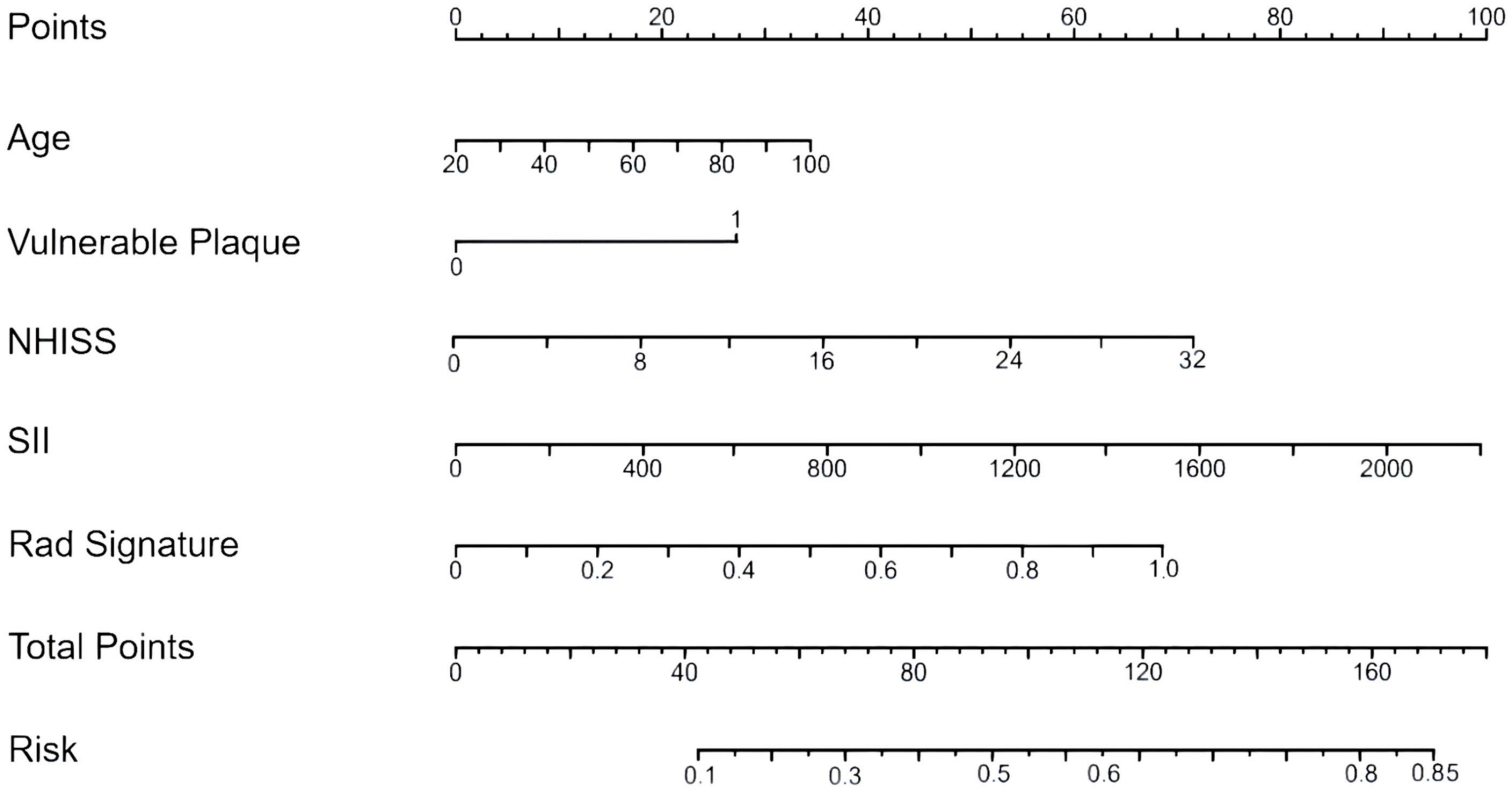
Ultrasonic radiomics column chart. In the training set, a chart encompassing both imaging features and clinical characteristics was constructed. The sum of scores for each variable yields a total score corresponding to the probability of predicting adverse outcomes. This allows for the direct assessment of whether each patient in the chart is at risk for adverse outcomes.

A radiomics nomogram was developed by integrating four clinical features with 11 ultrasound radiomic features (Figure 9). In the testing set, the model achieved a Brier score of 0.104, indicating a small deviation between predicted probabilities and actual outcomes. ROC curves comparing the clinical model, radiomic model, and radiomics nomogram. AUC values for three classifiers (LightGBM, LR, and KNN) based on the radiomic feature model in both training and testing sets. AUC values for the clinical model constructed with four clinical variables are provided in Supplementary Figure S1.

The radiomics nomogram yielded an AUC of 0.891 (95% CI: 0.832–0.950) in the training set and 0.852 (95% CI: 0.757–0.947) in the testing set. In the training set, the AUCs for the clinical model, radiomic model, and nomogram were 0.794, 0.879, and 0.891, respectively; in the testing set, the corresponding values were 0.765, 0.864, and 0.852 (Table 3). Accuracy, sensitivity, and specificity metrics demonstrated superior predictive performance for the radiomics nomogram. DeLong test results indicated statistically significant differences in AUCs between the nomogram and the other models in both sets (*p =* 0.05). The AUC values of the LR model were consistent across training and testing sets.

**Table 3:** Prediction efficiency indexes of the clinical signature, radiomic signature, and radiomic nomogram in the train and test set.

Signature	AUC	95% CI	Accuracy	Sensitivity	Specificity	PPV	NPV
Training group	0.794	0.707–0.881	0.781	0.728	0.642	0.703	0.648
Clinic Signature	0.879	0.817–0.941	0.825	0.723	0.790	0.796	0.726
Rad Signature	0.891	0.832–0.950	0.829	0.756	0.799	0.884	0.758
Testing group							
Clinic Signature	0.765	0.577–0.953	0.689	0.701	0.675	0.690	0.614
Rad Signature	0.864	0.766–0.962	0.774	0.785	0.818	0.798	0.736
Nomogram	0.852	0.757–0.947	0.789	0.805	0.777	0.889	0.762

AUC, area under curve; CI, confidence interval; PPV, positive predictive value; NPV, negative predictive value. After propensity score matching (*n* = 86), the radiomics signature maintained comparable predictive performance with AUC 0.861 (95%CI: 0.785–0.937). No significant differences remained in age (*p* = 0.32), NIHSS (*p* = 0.26), SII (*p* = 0.41) or vulnerable plaque prevalence (*p* = 0.19) between matched groups.

### The application value of clinical physicians

**Figure 10:**
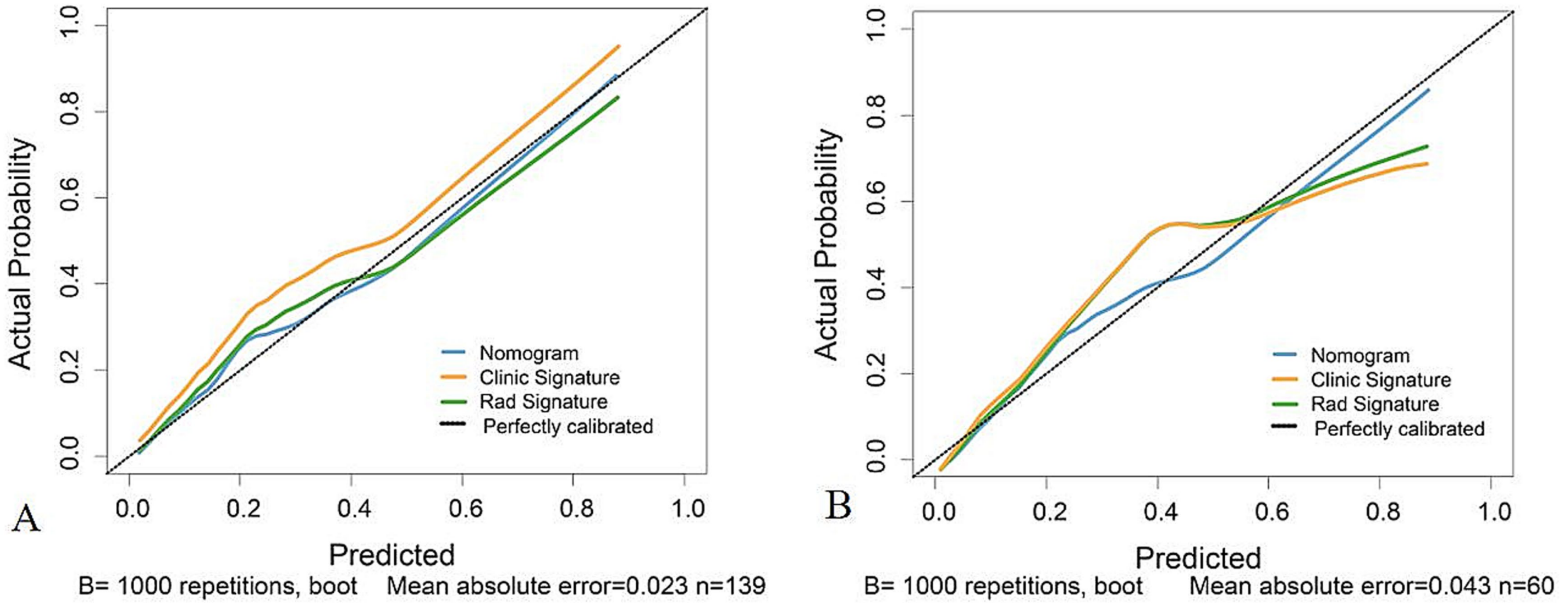
Calibration curves of different models. This figure displays the calibration curves of various models in both the training and testing datasets, showcasing the consistency between the predicted probabilities of adverse outcomes by the models and the actual observed probabilities. The calibration curves demonstrate a strong alignment between the predicted risks of adverse outcomes in stroke patients within 3 months and the observed risks in both the training and testing datasets.

A radionics-based prediction model was developed using carotid ultrasound images in AIS patients, and its performance was evaluated using multiple statistical methods. As shown in Figure 10, the calibration curve demonstrated good agreement between predicted probabilities and actual outcomes for adverse prognosis within 3 months. Model performance was consistent across training and testing datasets, with the Hosmer–Lemeshow test supporting its calibration. DCA in Supplementary Figure S2 indicated that the radiomics model yielded a higher net benefit than traditional clinical models across a range of threshold probabilities. Inter-observer agreement for plaque vulnerability assessment using CDU was high, with a Cohen’s kappa of 0.93 (95% CI: 0.87–0.97). Agreement for ulcerative plaques was 0.90 (95% CI: 0.81–0.97), for lipid necrotic core/intraplaque hemorrhage was 0.88 (95% CI: 0.80–0.96), and for fibrous cap rupture/incompleteness was 0.83 (95% CI: 0.74–0.94).

## Discussion

This study is the first to integrate CDU radiomic features with the SII and key clinical variables to construct a multimodal prediction model for assessing three-month outcomes in patients with AIS. Compared to high-resolution MRI, which has advantages in plaque composition analysis but is costly and time-consuming (28), our approach utilizes low-cost (approximately 50 RMB) and rapid (<10 min) CDU imaging to extract quantifiable high-dimensional radiomic features, significantly enhancing the identification of plaque heterogeneity and vulnerability (AUC = 0.876). This model offers strong predictive performance, practical utility, and high scalability, particularly for early risk stratification and precision management in low-resource settings.

The stratified analysis confirmed that age, NIHSS score, SII, and vulnerable plaques are independent predictors of poor prognosis at 3 months. Strict inclusion criteria were applied to improve data quality and model accuracy, limiting the cohort to patients admitted within 3 days of onset without severe comorbidities or atrial fibrillation. After propensity score matching, the model maintained strong predictive ability (AUC = 0.861), supporting its independence and complementary value to clinical variables. The model performed better in anterior circulation strokes, consistent with the anatomical role of carotid plaques in supplying the anterior territory, while the posterior circulation may be more affected by systemic inflammation. To further explain this difference, several physiological factors should be considered. The posterior circulation (vertebrobasilar system) is primarily supplied by the vertebral arteries rather than the carotid arteries. Embolic events resulting from carotid plaque rupture or shedding are more likely to affect the anterior circulation (middle cerebral artery and anterior cerebral artery territories), as the internal carotid artery directly supplies these regions. In contrast, posterior circulation ischemia involves more complex and heterogeneous pathological mechanisms, including perforator artery disease, vertebrobasilar dolichoectasia, and anomalies in vertebral artery origin, which are less directly related to carotid plaque characteristics. These factors may dilute the predictive value of carotid ultrasound radiomic features for posterior circulation strokes. Additionally, the relatively small number of posterior circulation cases in our cohort may have limited the statistical power to detect significant associations in this subgroup. This mechanistic heterogeneity reinforces the biological plausibility of the model. Previous studies have shown higher mortality and poorer recovery in elderly AIS patients, likely due to age-related decline and complications such as infections or hemorrhagic transformation (29, 30). The prognostic relevance of NIHSS and the role of inflammation in AIS progression have also been established (31). By excluding patients with atrial fibrillation, our study confirms that carotid plaque imaging features remain significant prognostic markers in non-cardioembolic stroke.

The SII, which integrates neutrophil, lymphocyte, and platelet counts, has been widely used to predict outcomes in various diseases (32). In AIS, neutrophils exacerbate brain injury by releasing pro-inflammatory mediators such as matrix metalloproteinases (33, 34), while regulatory T cells and certain T-cell subsets may exert neuroprotective effects through anti-inflammatory pathways. Platelet activation contributes to thrombosis and inflammation associated with cerebral ischemia (35, 36). In this study, SII demonstrated independent prognostic value as a global marker of inflammation.

Vulnerable carotid plaques also play a critical role in AIS progression, with previous studies indicating associations with prolonged hospitalization and poor functional recovery (37, 38). To eliminate confounding from baseline disability, patients with a modified Rankin Scale score greater than 2 were excluded, ensuring a more accurate assessment of plaque characteristics. Radiomics enabled the quantification of texture, grayscale, and morphological heterogeneity of plaques beyond visual assessment (38–40). Features like GLCM and GLRLM captured microstructural complexity and demonstrated a low correlation with subjective vulnerability scores, supporting their independent predictive value. The ROI was standardized to the largest longitudinal section of the plaque, with blind segmentation and inter-observer agreement testing to ensure feature stability (9). Mechanistically, metabolic disturbances and inflammation contribute to the formation of vulnerable plaques, which elevate the risk of thrombosis and cerebral ischemia, ultimately leading to poor AIS outcomes (41, 42). While several AIS prognostic models have been developed (43–45), many suffer from limitations in scoring systems or inconsistent variable definitions. By integrating radiomics with clinical and immunological markers, this study proposes a prognostic tool with enhanced biological plausibility and clinical applicability.

**Figure 11:**
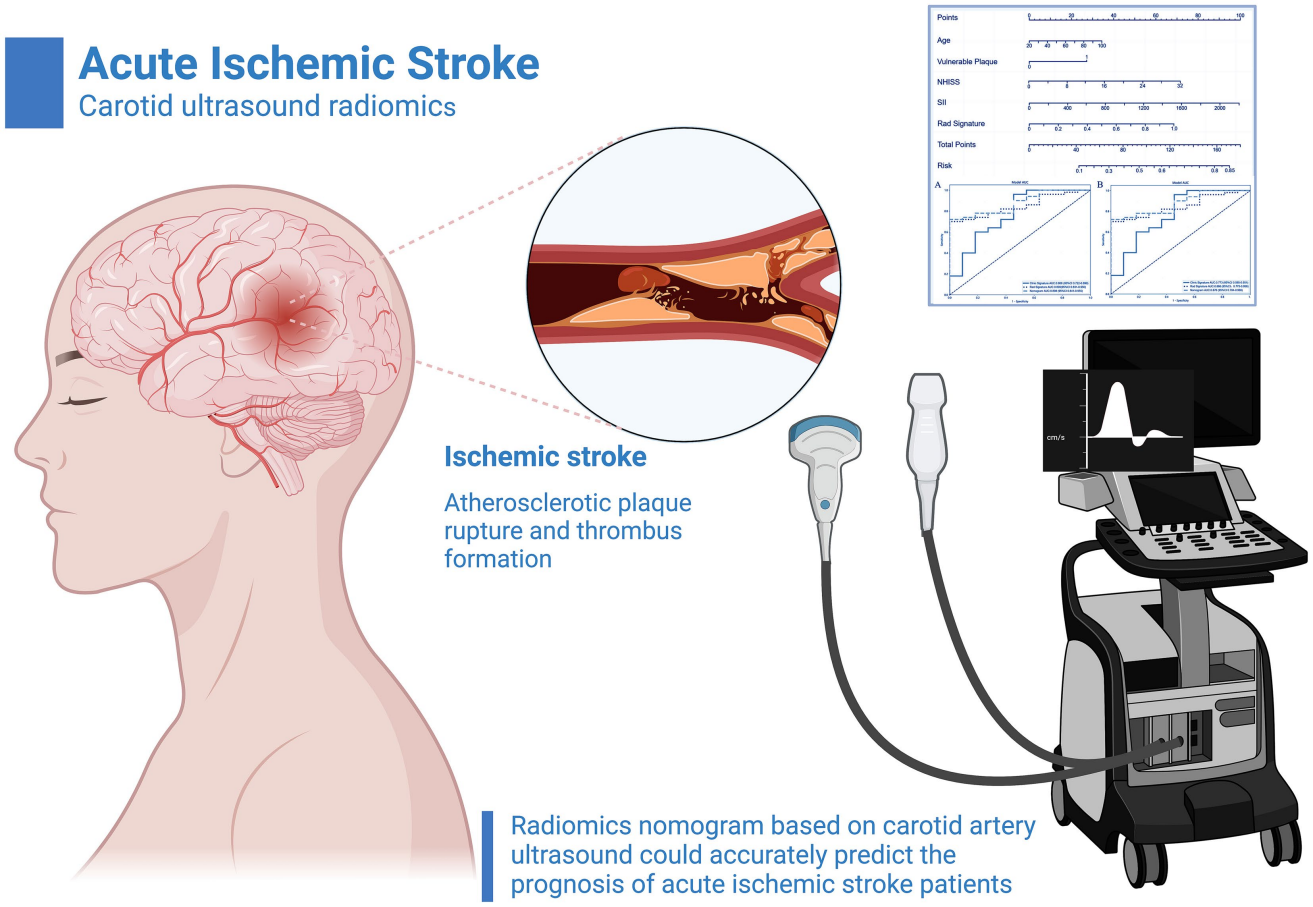
Analysis of radiomics and clinical factors in prognosis of AIS patients.

Previous studies have developed prognostic scoring systems for AIS using multivariate logistic regression. For example, the Acute Stroke Registry and Lausanne Score identified age, NIHSS score, time from onset to admission, visual field deficits, blood glucose, and level of consciousness as key variables (45). However, some of these predictors rely on subjective assessment or are unsuitable for patients with impaired consciousness, limiting their generalizability. In contrast, our model incorporates the modified Rankin Scale (mRS), NIHSS, SII and radiomic features derived from CDU, offering a more clinically adaptable tool. NIHSS reflects the severity of neurological deficits, and SII captures the systemic inflammatory burden. Together with plaque imaging features such as ulceration or irregular borders, these variables provide a multidimensional framework for outcome prediction. Integrating radiomic nomograms and conventional clinical indicators demonstrated superior performance and practical potential compared to traditional scoring systems (Figure 11).

Regarding model development, LASSO regression was used for feature selection, followed by model construction and comparison using three machine learning algorithms: LightGBM, LR, and KNN. LR achieved the best performance in the radiomics model, combining high training efficiency with interpretability and the ability to quantify the impact of predictors through odds ratios, making it highly suitable for clinical application (46). Although KNN is advantageous for nonlinear data, it is highly sensitive to class imbalance, which likely contributed to its suboptimal performance in this study. Overall, the CDU-based radiomics model outperformed other methods in training and testing cohorts and demonstrated good calibration consistency, supporting its reliability and robustness in AIS prognostic assessment (47).

Despite the innovative nature and clinical applicability of this study, several limitations should be acknowledged. First, as a prospective cohort with retrospective radiomic analysis, there is a potential risk of selection bias. Second, the relatively small sample size may limit the generalizability of the model. Incomplete acquisition of key imaging data, such as infarct location, volume, and collateral circulation status, may affect predictive performance across varying lesion characteristics. The retrospective nature of the radiomic analysis (i.e., feature extraction and model development performed on prospectively collected data) also limited the evaluation of dynamic infarct evolution, such as 24-h volume expansion, which future studies should address using longitudinal radiomic data.

Plaque vulnerability scores in this study were inferred primarily from imaging features. Although double-blind segmentation and inter-observer agreement assessments were conducted to reduce subjectivity, further histopathological validation is warranted to enhance accuracy. Additionally, external validation using independent cohorts is lacking.

Furthermore, although our subgroup analysis showed better performance in anterior circulation strokes, the inclusion of both anterior and posterior circulation cases in the primary model may have introduced some heterogeneity.

Future research should include multicenter, prospective studies to systematically assess model performance across diverse populations and imaging platforms. Expanding the sample size and refining feature selection and modeling algorithms will improve prediction accuracy and facilitate broader clinical implementation of AIS prognostic tools.

## Data Availability

The raw data supporting the conclusions of this article will be made available by the authors, without undue reservation.

## Supplementary material

SUPPLEMENTARY FIGURE S1 ROC curves of the clinical model in the training and testing sets. Area under the curve (AUC).

SUPPLEMENTARY FIGURE S2 DCA of clinical features, radiomic features, and radiomic nomogram. This figure illustrates the DCA results of clinical features, radiomic features, and radiomic nomograms, comparing the efficacy of these models in clinical decision-making. In comparison to scenarios without using any predictive models (all-treatment or no-treatment strategies), a radiomic nomogram demonstrates significant intervention benefits in patients with predictive probabilities, as opposed to clinical features and radiomic features. DCA stands for decision curve analysis.
