# Supplementary material for: Advanced radiomics techniques applied on carotid duplex ultrasound data predict adverse outcomes in acute ischemic stroke
Source: Front Neurol. 2026 Aug 5;17:1878428. doi: 10.3389/fneur.2026.1878428 (PMC13487531; doi:10.3389/fneur.2026.1878428)
Supplement: Supplementary file 1 [file Supplementary_file_1.PDF]

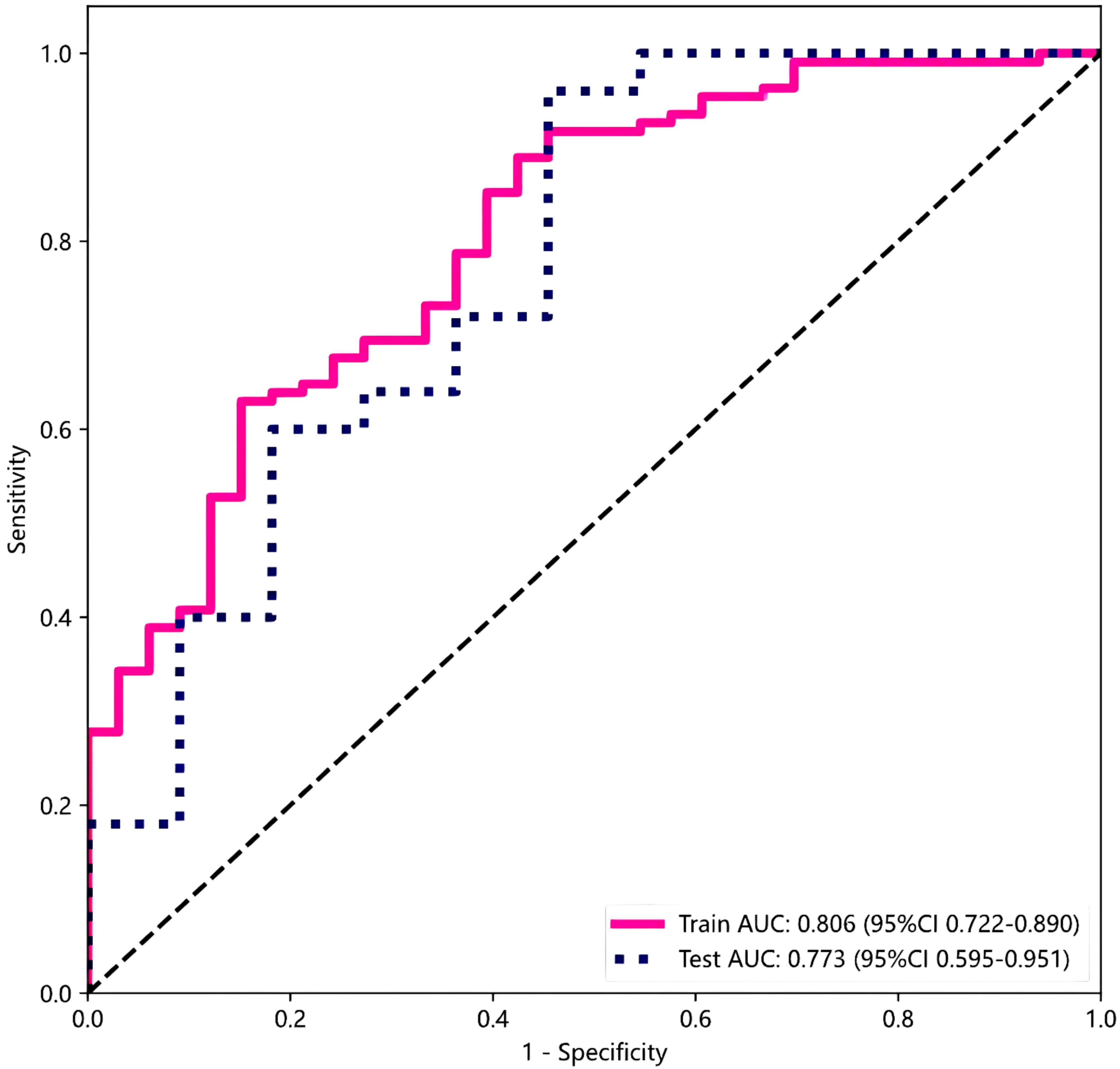

Sensitivity
1.0
0.8
0.6
0.4
0.2
0.0
0.0
0.2
0.4
0.6
0.8
1.0
1 - Specificity
Train AUC: 0.806 (95%CI 0.722-0.890)
Test AUC: 0.773 (95%CI 0.595-0.951)
