# Supplementary material for: Advanced radiomics techniques applied on carotid duplex ultrasound data predict adverse outcomes in acute ischemic stroke
Source: Front Neurol. 2026 Aug 5;17:1878428. doi: 10.3389/fneur.2026.1878428 (PMC13487531; doi:10.3389/fneur.2026.1878428)
Supplement: Supplementary file 2 [file Supplementary_file_2.PDF]

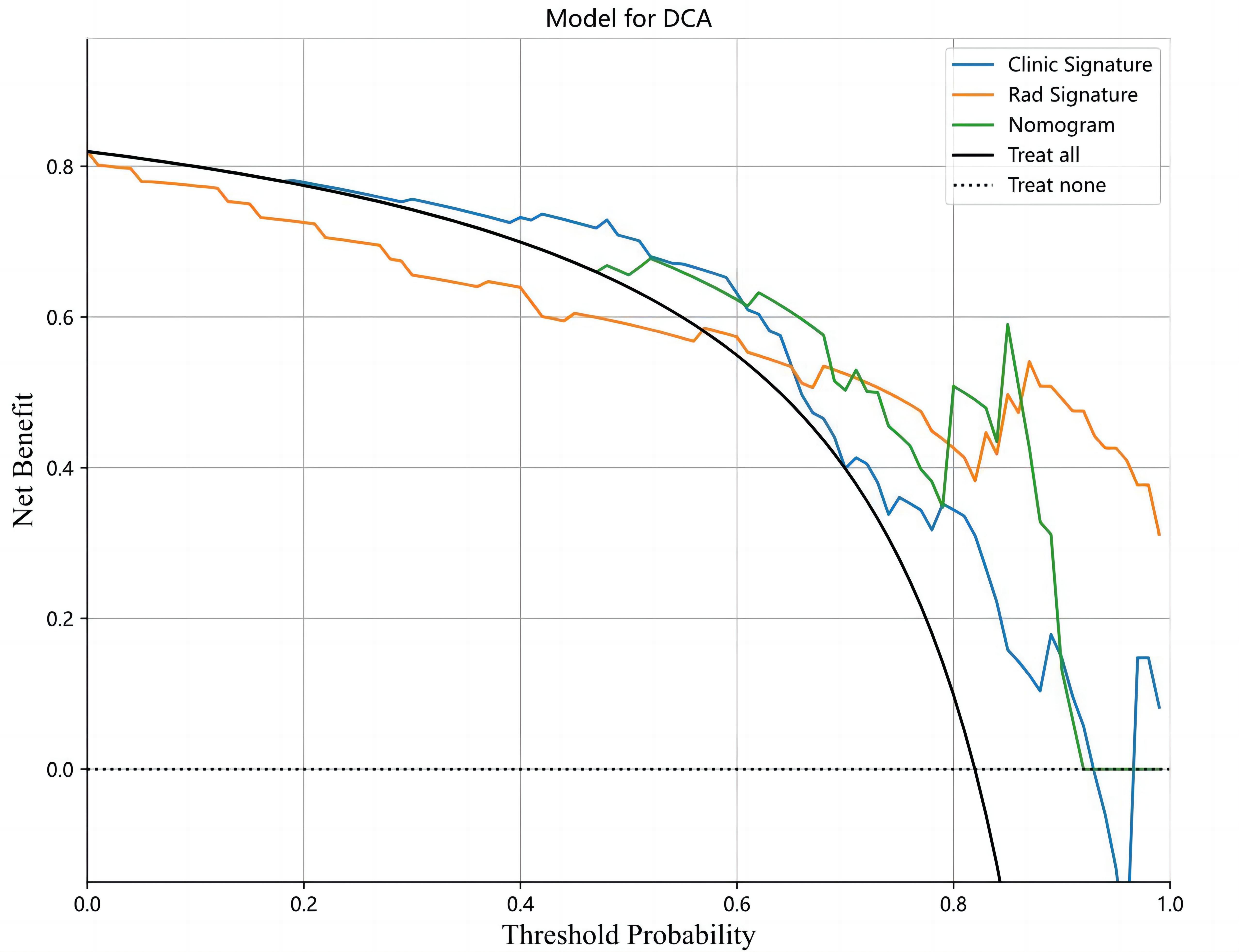

Model for DCA
Clinic Signature
Rad Signature
Nomogram
Treat all
Treat none
Net Benefit
0.8
0.6
0.4
0.2
0.0
0.0
0.2
0.4
0.6
0.8
1.0
Threshold Probability
